# The Bioassay-Guided Fractionation and Identification of Potent Acetylcholinesterase Inhibitors from *Narcissus c.v. ‘Hawera’* Using Optimized Vacuum Liquid Chromatography, High Resolution Mass Spectrometry and Bioautography

**DOI:** 10.3390/metabo10100395

**Published:** 2020-10-04

**Authors:** Tomasz Mroczek, Aleksandra Dymek, Jarosław Widelski, Krzysztof Kamil Wojtanowski

**Affiliations:** Independent Laboratory of Chemistry of Natural Products, Chair of Pharmacognosy, Medical University, 1 Chodzki St., 20-093 Lublin, Poland; aleksandra.dymek91@interia.pl (A.D.); yarpen222@interia.pl (J.W.); krzysztofkamilw@gmail.com (K.K.W.)

**Keywords:** *Narcissus triandrus L. c.v. ‘Hawera’*, amaryllidaceae alkaloids, vacuum liquid chromatography, TLC-bioautography, HPLC/ESI-QTOF-MS, AChE inhibitors

## Abstract

Bioassay-guided isolation of bioactive compound is a modern and efficient technique in metabolites screening. It may shorten the total time of the entire process and reduce some costs of it. The aim of this paper was to fractionate and isolate alkaloids by developing an innovative vacuum liquid chromatography method for a species of *Narcissus c.v. ‘Hawera’* rarely investigated so far and establishing the inhibitory activity of acetylcholinesterase (AChE). The studies consisted of the extraction of plant material by modern pressurized liquid extraction (PLE), followed by the isolation of alkaloidal fractions. For this purpose, the pioneering gradient vacuum liquid chromatography (gVLC) technique was employed by using two sorbents in various proportions packed in polypropylene cartridges for the first time. This step was performed in order to pre-clean the samples but also to establish the best combination of sorbents which permits obtaining potentially strong AChE inhibitors. The collected fractions were examined by HPLC/ESI-QTOF-MS in order to compare which combination of sorbents would allow us to obtain the highest concentration of alkaloids. The combination of these techniques confirmed the presence of the alkaloids and enabled the development of a modern method for the fractionation and isolation of the compounds with strong anti-AChE activity.

## 1. Introduction

Nature provides a huge diversity of compounds, which can be used for example as anticancer agents (curcuminoids from *Curcuma longa*) [1] or as a source of antioxidant polyphenolic ingredients of functional food (e.g., chokeberry-*Aronia melanocarpa*) [2]. Many of the active constituents encountered in plants show different activity on central nervous system (CNS), i.e., coumarins which can act as anticonvulsant, antidepressant, anxiolytic, precognitive or neuroprotective agents [3,4,5,6].

**Figure 1:**
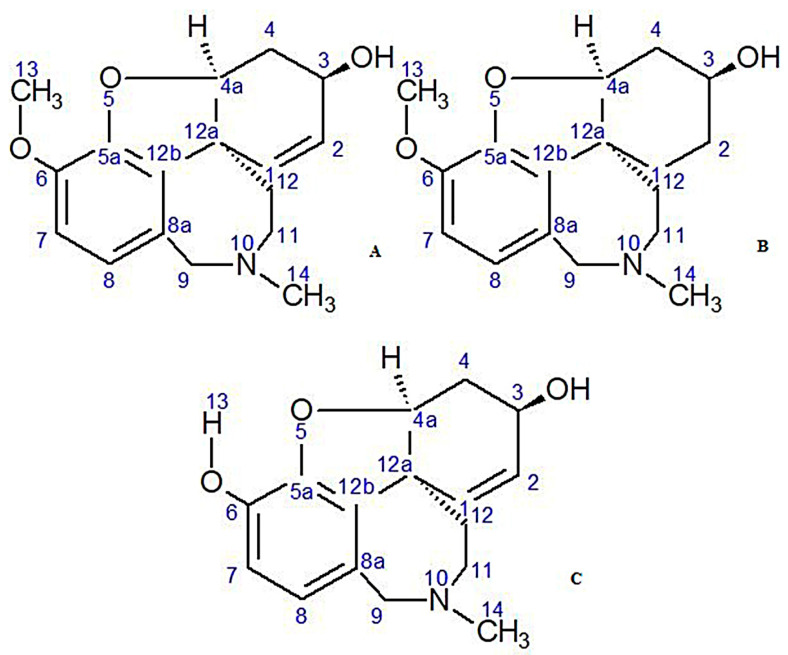
Chemical structures of galanthamine (**A**), lycoramine (**B**) and sanguinine (**C**)-as strong AChE inhibitors detected.

Alkaloids are the group of substances occurring often in nature both in plant and animal kingdoms. They play very important role mainly due to their strong antioxidant, anxiolytic and antidepressant properties [7]. They have been also used in the treatment of neurodegenerative diseases [7]. Plants of the Amaryllidaceae family, particularly those belonging to species of *Narcissus*, are a rich source of alkaloids. The alkaloids are mostly present in the bulbs and flowering parts [8,9]. High concentration of alkaloids, including lycorine, which was first isolated from these plants, and crinine, augustamine or galanthamine, which is used in the treatment of neurodegenerative illnesses such as Alzheimer’s disease (AD) (Figure 1), has been found. These compounds exhibit even wider range of bioactivities including anticancer, antiviral, antibacterial, antifungal, analgesic, cytotoxic and acetylcholinesterase (AChE) inhibitory activities [8,9]. They have been used since antiquity [10]. AD being the main cause of dementia in the contemporary world is characterized by acetylcholine (ACh) decrease in cholinergic neurons. Therefore, an increase the levels of ACh by inhibition of this ACh-hydrolyzing enzyme, is very important. Galanthamine, the main alkaloid found in this family, acts by inhibiting both the enzymes: AChE and butyrylcholinesterase (BuChE), which leads to the increase in the concentration of ACh in the synapses of the CNS. Cholinesterase inhibitors are currently the only available medicinal therapies used to treat patients suffering from mild to moderate AD. The therapy involves only stopping of the symptoms of the disease, through intensification on neurotransmission in the cholinergic system. Therefore, many attempts are being made to search for AChE inhibitors with greater efficiency and better pharmacokinetics [11]. Searching for natural inhibitors of different enzymes, e.g., AChE, is also a modern approach in new drug discovery [9].

*Narcissus* species belonging to the Amaryllidaceae family are also very rich source of alkaloids which are potent, reversible and highly selective AChE inhibitors. Our investigated species, *Narcissus triandrus L. c.v. ‘Hawera’*, has been rarely tested for the inhibitory activity of AChE so far. No data on the isolation of the alkaloids from this plant material are available in the literature. For this purpose, dried bulbs from *Narcissus ‘Hawera’* have been investigated in order to find and isolate AChE inhibitors and also to compare their composition with other plants belonging to Amaryllidaceae family. Extraction of alkaloids was performed through PLE (Pressurized Liquid Extraction) at elevated temperatures and pressure using methanol (MeOH) as polar solvent. Thanks to this automated method, a large number of extracts needed for further experiments has been quickly obtained. The choice of the method of alkaloids isolation was a big challenge. According to the attempts to isolate active compounds that have been carried out so far, we concluded that the Vacuum Liquid Chromatography (VLC) method may be an effective tool. The first mention of VLC appeared in the second half of the 20th century, but it was described in more detail in 1979 by Targett et al. This method has been classified as a preparative liquid chromatography [12,13,14,15]. For many years, it has been used to isolate not only the alkaloids but also other natural compounds from plant extracts due to the simplicity of its preparation and operation. Modification of this method through the ability to select the appropriate sorbent and eluent allowed one to obtain quite effective purification of the samples in some works [16,17] but, on the other hand, no method using sorbent gradients has been so far developed and described.

In one of the tests, two fast column chromatography techniques: flash chromatography and vacuum liquid chromatography for separation of the pungent principles (gingerols and shogaols) of the ginger powder extract, were compared. The results obtained confirmed the effectiveness of the VLC method. The main gingerol homologues were satisfactorily separated from the other compounds only using VLC method. It was reported that this method has a number of advantages, e.g., reduction of production costs and time of experience [18]. The equipment is cheap and available in most laboratories, and the solvent system is easy to develop by mixing it with different polarities and concentrations [18]. Pelletier came to similar conclusions in his work, showing the superiority of the VLC method compared to conventional preparative layer chromatography (PLC) [19]. This latter technique has proven to be tedious, time-consuming, expensive and useful for small-scale separation only.

Knowing possible advantages of VLC in a rapid separation and fractionation of natural compounds we decided to develop bioassay guided fractionation of the alkaloids from the bulbs of *Narcissus triandrus L. c.v. ‘Hawera’* using an optimized and a novel gradient VLC approach and find the best experimental conditions for an efficient isolation of alkaloids with strong anti-AChE activity. To date, only few papers on VLC isolation of Amaryllidaceae alkaloids have been published [20,21], but none of them described comprehensive combination of PLC, gradient VLC, TLC-bioautography and HPLC/ESI-QTOF-MS for rapid screening and fractionation of the bioactive Amaryllidaceae alkaloids. Bioassay-guided isolation of bioactive compound is a modern and efficient technique in metabolites screening. It may shorten the total time of the entire process and reduce some costs of it. In some of our published papers we postulated that both TLC-bioautographic techniques and hyphenated chromatographic ones with a high resolution mass spectrometry can be used for the analytical scale studies [22,23,24]. Now, based on this paper, we also tried to extend them in more preparative scale.

Moreover, for the visualization of AChE activity, we conducted tests using a modern automated device which replaced the technique of manual spraying of TLC plates. This derivatizer is a kind of chamber which sets a new standard of reproducibility and convenience in reagents transfer onto TLC plates. In our experiments presented here AChE inhibitors were isolated and identified based on the assay elaborated at our laboratory. In some fractions even a small percentage of galanthamine, was observed. As mentioned above it increases ACh concentration in synapses of CNS [24,25,26,27]. The first published work on inhibition of AChE by galanthamine appeared thanks to Maskowski and Kruglikov-Lvov in 1951. It was first isolated from the leaves and flowers of the *Galanthus woronowii* by the Bulgarian scientist Paskov in 1959, who also proved galanthamine’s AChE inhibiting properties and even neuromuscular conduction [22,28,29]. Thanks to the developed here procedure, AChE inhibitory activity was also confirmed by many of other alkaloids present in isolated fractions.

## 2. Results and Discussion

### 2.1. Extraction and Optimization of Fractionation by VLC for the Isolation of Alkaloids from Narcissus triandrus L. c.v. ‘Hawera’

Establishing appropriate conditions for PLE extraction and by the use of MeOH as the polar solvent we were able to obtain three concentrated extracts and prepare them for subsequent analysis. The extraction process was carried out at high pressure and temperature using small amounts of solvent. Its advantages also included: automatization of the extraction process and shortening of the extraction time. The samples prepared in this way were subjected to further purification processes.

The fractionations were carried out by the innovative gradient VLC methods, with a proper combination of the sorbents which allowed us to obtain higher rates of analytes recoveries and improved separation of the alkaloidal fractions. Such the sorbent gradients have been applied in VLC separation of *Narcisssus* alkaloids for the first time. There was a question about the type of sorbents used to fill the columns in order to achieve a good performance of the entire process. We decided to apply a mixture of two the most popular polar sorbents, as a technique which has not been used in VLC process of these alkaloids so far. Therefore, the concentrated methanolic extracts have been transferred into glass column or polypropylene cartridge filled with different combinations of two polar sorbents: silica gel 60 F_254_ and basic aluminum oxide (alumina–Al_2_O_3_) (150 MeSh) as two-stationary phase systems in various proportions and different order aiming to isolate alkaloidal fractions. These columns were prepared by uniform packing using appropriate sorbents in a vacuum. In contrast to other column chromatography techniques, a vacuum was used to increase the flow rate of fractionation procedure.

These 3 experiments were aimed at comparing the type of sorbents used as well as the kind of columns used to obtain the alkaloidal fractions with higher bioactivity. The first experiment using the glass column filled with silica gel and Al_2_O_3_ in 1:1 ratio turned out to be a tedious and time-consuming method, where it was necessary to pack a relatively long column with a large amount of sorbents. The process of conditioning and saturation of sorbents was much longer compared to other two experiments. The remaining two further VLC methods were much more efficient and less time consuming. In these methods one polypropylene cartridge was used and alternately filled with Al_2_O_3_ and silica gel in volume ratios of 1:3 or 3:1. The elution step was the same for each of the three VLC techniques. Due to different polarity of individual alkaloids, gradient elution was necessary. Therefore, the proper selection of the mobile phase gradient system and its modification by adding MeOH solution and small amounts of 25% aqueous ammonia solution was necessary. It enabled the separation of alkaloids depending on their polarity in a relatively short time. Hydrophobic compounds were obtained in the first fractions due to the polar nature of the sorbents, and the choice of less polar mobile phase. For the elution of very polar alkaloids such as sanguinine, from the stationary phase, a 100 mL solution of CHCl_3_:MeOH (1:1, *v/v*) was used, followed by 50 mL of MeOH alone. The alkaloids of *Narcissus ‘Hawera’* were eluted at a rate depending on polarity of their moieties, starting from nonpolar alkaloids and ending with sanguinine and further polar alkaloids. The obtained fractions were analyzed by TLC method on silica gel plates with four standards: sanguinine, lycoramine, *nor*-galanthamine and galanthamine used for comparison and confirmation of the presence of the alkaloids. The preliminary TLC results confirmed the presence of these four alkaloids in the obtained fractions and showed the existence of new, unidentified ones. Based on the TLC profile, identical fractions were collected together, and initial fractions with red fluorescence under UV light were discarded, showing no alkaloids but confirming the presence of chlorophyll. The obtained and combined fractions from these three experiments (30 in total) are shown in Table 1.

The separated alkaloids were visualized under UV light. The developed methods allowed us to separate individual components and to confirm the presence of almost pure sanguinine fractions. They were useful for fractionation of natural products (sometimes as the only possible way of isolation) before subsequent analysis steps, such as the High Performance Liquid Chromatography (HPLC) method. The next stage of research included the identification of alkaloid compounds by High Performance Liquid Chromatography/Electrospray-Ionization-Time-of-Flight-Mass Spectrometry (HPLC/ESI-QTOF-MS), followed by TLC-bioautographic approach towards inhibition of acetylcholinesterase enzyme.

### 2.2. LC-MS Identification of the Isolated Compounds

The purified samples were analyzed using the HPLC/ESI-QTOF-MS system. This is an efficient technique which is used to determine unknown compounds present in the studied plant extracts, as it was documented in our previous papers [23,24]. It combines chromatographic separation of plant material with its analysis by high resolution mass spectrometry.

This method allowed us to separate a mixture containing different compounds based on HILIC retention. After the separation, the compounds were subjected to further analysis using the ESI-QTOF-MS spectrometer in positive ion mode. Thanks to this method, high resolution MS spectra were obtained and in combination with TLC-bioautography enabled structural determination of the most active AChE inhibitors in these individual fractions. The active alkaloids obtained in these three experiments are included in the Tables (Table 2, Table 3 and Table 4). Their determination and identification was based on high mass accuracy CID spectra and analysis of the protonated molecules fragmentation pathways. In general, mass error was below 1 ppm. The alkaloids characteristic of the Amaryllidaceae family were not isolated in all collected fractions. Therefore, some fractions were omitted in subsequent results.

Until now, over 300 alkaloids from the Amaryllidaceae family have been isolated, including up to 100 from the *Narcissus* genus [8]. High amounts of the alkaloids have been identified in the *Narcissus c.v. ‘Hawera’* species tested, some of which dominated and others in trace amounts only. A total of 13 alkaloids structurally related to AAs were detected. The main ones detected in this species were as follows: lycorine, lycoramine, sanguinine, ungeremine, galanthamine, 4,*N-*didehydro-*nor*-augustamine and others.

Eight alkaloids were isolated in the first VLC experiment at various concentrations in terms of their amount to the total amount of alkaloids in a given fraction based on analysis of the TIC chromatograms of that fraction (Table 2). Lycorine was the major alkaloid present in each sample (A-19-34) and it constituted high percentage of the samples. This compound is one of the most abundant alkaloids commonly found in the Amaryllidaceae family. In the first investigated fraction (A-19), the percentage of lycorine was small, but in subsequent sample it considerably increased and even constituted half of the analyzed mixture. Lycoramine (dihydrogalanthamine) was also isolated in a large percentage of the samples (A-19-22 and A-26-34).

**Figure 2:**
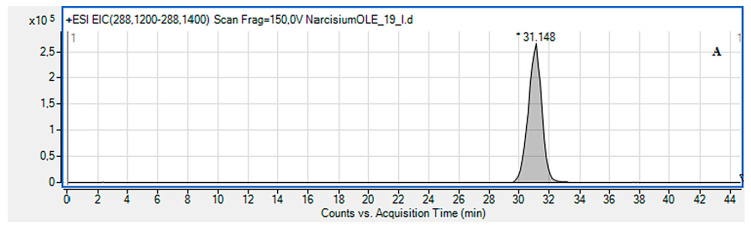

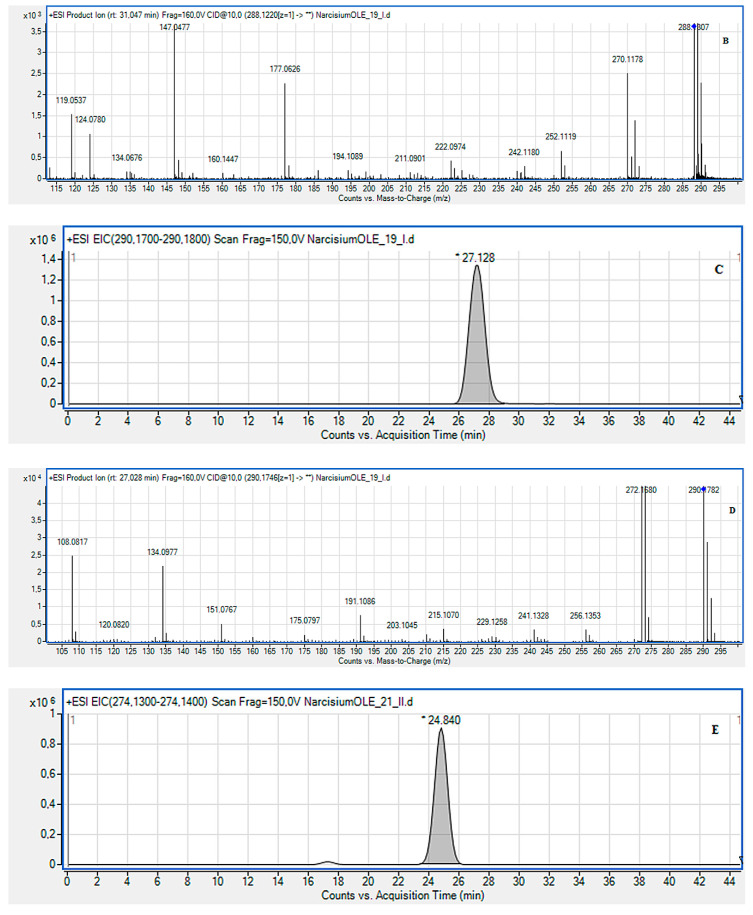

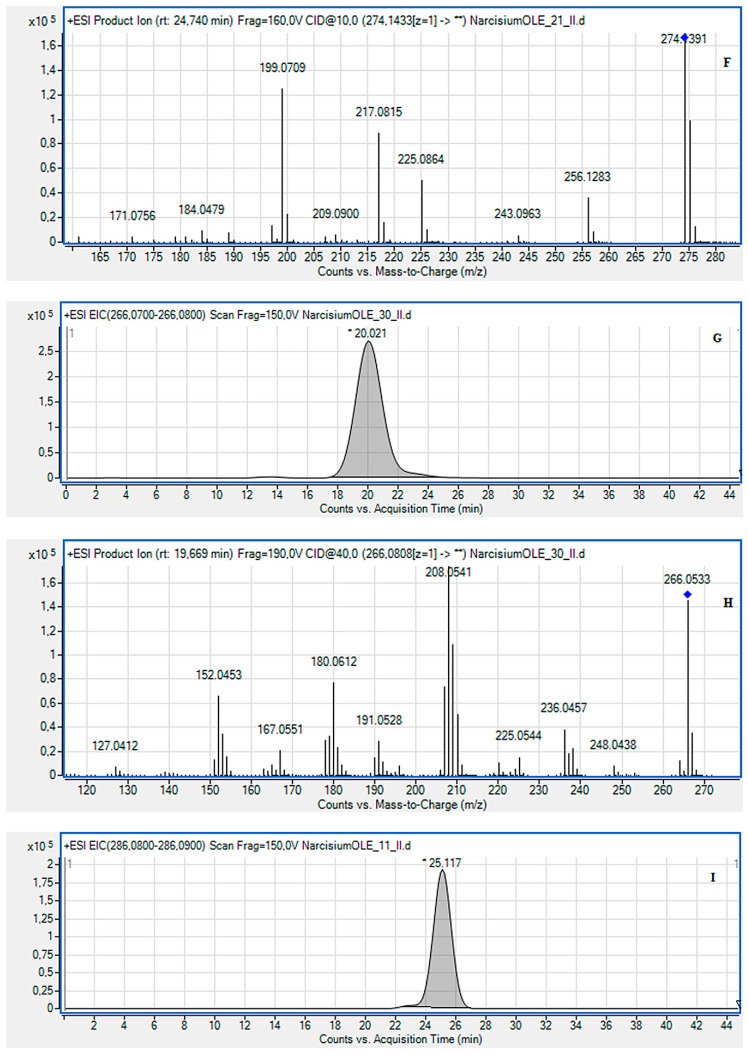
Extracted ion chromatogram (EIC) of *Narcissus Hawera* determined by HPLC/ESI-QTOF-MS in range of *m/z* 288.12-288.14 showing the lycorine (**A**); lycoramine (**C**) at *m/z* 290.17-290.18, and sanguinine (**E**) at *m/z* 274.13-274.14 identified in obtained fractions. Below high-quality peak spectra show strong AChE inhibitors with the highest percentage of their occurrence in the fractions. CID MS/MS product ion spectra using 10 eV of collision energy were performed by HPLC/ESI-QTOF-MS to determine: lycorine (**B**) lycoramine (**D**), and sanguinine (**F**) identified in the obtained fractions. Axis Y shows peak intensity. EIC determined by HPLC/ESI-QTOF-MS in range at *m/z* 266.07–266.08 showing ungeremine (**G**); 4,*N*-didehydro-*nor*-augustamine (**I**) at *m/z* 286.08-286.09, and tetrahydro-*nor*-augustamine (**K**) at *m/z* 284.098–284.099 identified in obtained fractions. CID MS/MS product ion spectra using 40 eV of collision energy were performed by HPLC/ESI-QTOF-MS to determine: ungeremine (**H**), 4,*N*-didehydro-*nor*-augustamine (**J**), and tetrahydro-*nor*-augustamine (**L**) as the most commonly found alkaloids in the obtained fractions. Axis Y shows peak intensity.

In these fractions lycorine and lycoramine have been also identified based on the occurrence of the characteristic fragment ions (Figure 2A–D the protonated molecules of lycorine and lycoramine are shown with their fragmentations). For the peak at Rt = 31.15 min of the protonated molecule equal to *m/z* 288.1222 assigned to lycorine with *m/z*: 270.1187, 177.0626 and 147.0477 fragments, the presence this alkaloid (CID, 10 eV) has been confirmed. Another peak at Rt = 27.13 min for the protonated molecule at *m/z* 290.1746 was assigned to lycoramine, where product ions at *m/z* 272.1680, 256.1353 and 215.1070 supported the existence of this compound (CID, 10 eV). The others alkaloids were identified in the similar way. In the final fractions (A-26, A-27-33), even a small percentage of galanthamine was observed. Compounds belonging to the *nor-*augustamine derivative: 4,*N*-didehydro-*nor*-augustamine and tetrahydro-*nor*-augustamine were present in high concentration in the final fractions (A-26, A-27-33) (Table 2).

In the second experiment by using the cartridge and different amounts of the sorbents, the percentage of these three alkaloids—lycorine, lycoramine and galanthamine—was clearly determined. The first three fractions were omitted, because the analyzed MS spectra did not correspond to the alkaloids of the Amaryllidaceae family (Table 3). The use of one polypropylene cartridge filled with three parts of silica gel and one part Al_2_O_3_ allowed us to obtain more fractions (15 in total B3-B31) and to identify more alkaloids by LC-MS method in the second experiment. Cleaner fractions with higher percentage of the dominant alkaloid were also observed. In this experiment the presence of sanguinine was clearly observed in subsequent fractions (B15-26, B30-31). The previous papers found out that sanguinine is one of the major alkaloid of the Amaryllidaceae family, with stronger AChE inhibitory properties than galanthamine [9]. This fact is being explained by the presence of an additional hydroxyl group of sanguinine at the ring [9,20]. Ungeremine, 4, *N-*didehydro-*nor*-augustamine, tetrahydro-*nor*-augustamine were three alkaloids isolated from this plant species, which occurred in a high percentage in each study (Table 2, Table 3 and Table 4).

The identification of the alkaloids present in the fractions from the second experiment was performed in similar way as for the first experiment. All the compounds had the characteristic high resolution mass-to-charge ratios for [M + H]^+^ or M^+^ ions. From the analysis of the CID fragmentation pathways, a given type of structure could be established. For the example, a peak in extracted ion chromatogram (EIC) at *m/z* 274.13–274.14 was characteristic for sanguinine, whereas that one at *m/z* 266.07–266.08 was for ungeremine. The EIC and CID MS/MS spectrum of the sanguinine peak at Rt = 24.84 are shown in Figure 2E,F. The protonated molecule equal to *m/z* 274.1391 and *m/z* 256.1283, 217.0815, 199.0709 as the fragment ions confirmed its presence. Similarly, ungeremine with Rt = 20.02, (Figure 2G–L) corresponds to the protonated molecule at *m/z* 266.0808 and the fragment peaks: at *m/z* 266.0533, 248.0438 and 208.0541. The conducted research showed that compounds belonging to augustamine derivatives constitute a high percentage in each of those three experiments. Therefore, *Narcissus ‘Hawera’* bulbs can be considered a good source of these compounds.

The third method used one polypropylene cartridge filled with one part of silica gel and three parts of Al_2_O_3_. Eleven active alkaloids from ten collected plant fractions (C2-25) were separated and identified. Decreased concentration of sanguinine has been noticed as compared with the first experiment. In addition, galanthamine was found only in trace amounts. Similarly to other experiments, a high percentage of lycorine and ungeremine was detected (Table 4**)**.

### 2.3. TLC-Bioautography for the Detection of Potent AChE Inhibitors

**Figure 3:**
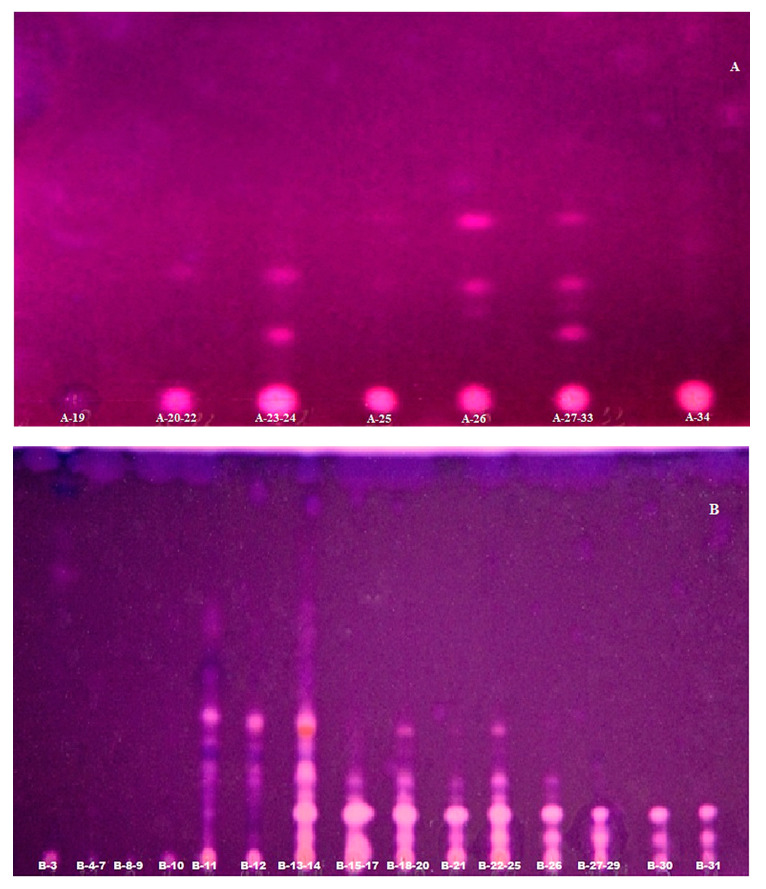

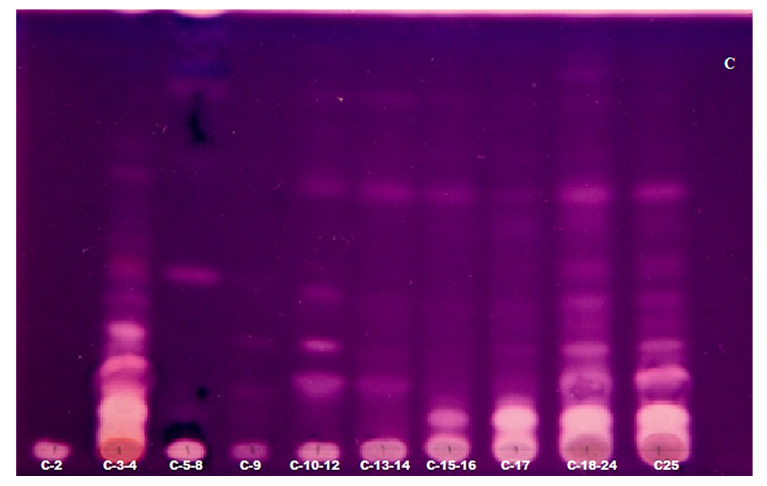
Videoscans of TLC-bioautographic anti-AChE activity performed by the modified Fast Blue B Salt method showing the fractions obtained from three gVLC experiments. (**A**) A19–A34 fractions obtained in the first experiment consisting of one glass column filled with equal amounts of Al_2_O_3_ (150 MeSh) and silica gel (60 F254); (**B**) B3–B31 fractions obtained in the second experiment consisting of polypropylene cartridge filled silica gel and Al_2_O_3_ in 3:1 ratio; (**C**) C2–C25 fractions obtained in the third experiment consisting of polypropylene cartridge filled with silica gel and Al_2_O_3_ in 1:3 ratio.

The first step was to use an ethyl acetate substrate added directly to the mobile phase. The plates with applied fractions were developed using vertical chamber because better separation results were obtained, compared to horizontal chamber. As a result of this enzymatic reaction, the product 2-naphthol was obtained. The next stages of this method have been refined by using a modern device, such as Camag Derivatizer. In these experiments, the use of this equipment made it an innovative method. This automated spraying technology has become useful for applying the reagent to plates and obtaining more reproducible results (three videosan A–C are presented in Figure 3). Reagent consumption is definitely less than using a manual spray method. In addition, four spray nozzles are available that allow you to meet the diverging physicochemical properties of the different reagents (e.g., viscosity or acidity). As a result, it was possible to use the enzyme–AChE in sequence to induce the reaction and the Fast Blue B Salt reagent to visualize zones and inhibitors. Three plates from three experiments were obtained showing the separation of the analyzed fractions, which allowed us to detect the most active AChE inhibitors. The deep purple background of the plate was obtained as a consequence of the reaction of 2-naphtol with Fast Blue B Salt reagent, and white spots indicated the presence of AChE inhibitors, which are stable for a long time [20,21]. Strong enzymatic activity represented as white spots on a purple background can be seen especially in the second and third experiments (videosan B–C are presented in Figure 3). The results obtained were compared with four available standards, which confirmed the presence of AAs and AChE inhibitors. Moreover, it was also supported by the HPLC/ESI-QTOF analysis. The resulting chromatograms of the peaks were identified based on the mass to charge ratio and by analyzing of CID MS/MS spectra of the protonated molecules as it was described above. Based on these results, we can assume that the white spots of the inhibitors might be the following alkaloids: lycorine, ungeremine, mesembrinol and 4,*N*-didehydro-*nor*-augustamine, tetrahydro-*nor*-augustamine, which occur in a large percentage in each of the studies performed (Table 2, Table 3 and Table 4). Moreover, the tests showed that compounds belonging to sanguinine and the augustamine derivatives are highly active. Their high percentage occurs in each of the three experiments. Therefore, *Narcissus ‘Hawera’* bulbs can be considered a good source of these compounds. Moreover, TLC-bioautography using the automated modern device is considered a fast qualitative tool for detecting compounds with anti-AChE activity in plant fractions. The operation of the chamber is simple and the results obtained are reproducible and independent with considerable reduction of expensive reagents, especially the enzymes. The total analysis time has been reduced from 2 h to 1 h, including the plates development and automated detection of the inhibitors. The usage of the expensive enzyme has been reduced two times. In addition, this method is safe for the analyst and the environment, thanks to safe handling through a closed system. The accuracy of the results obtained and the effect far exceed the manual method of spraying. In many articles, the TLC-bioautographic methods confirmed the antibacterial, antifungal and enzymatic inhibition of the investigated samples [25] more rapidly, with lower detection limits than in other bioassays [24].

A more accurate qualitative separation and visibility of individual bands were observed in the third experiment. This may indicate better purification of the fraction and separation of alkaloids by using polypropylene cartridge and choosing a compatible solid phase (videoscan C in Figure 3).

## 3. Materials and Methods

### 3.1. Plant Material

The plant material used for research was a *Narcissus triandrus L. c.v. ‘Hawera’* species belonging to Amaryllidaceae family. The plant specimen was purchased from Florexpol in Lublin, with the appropriate certificates of authenticity. The samples of this plant are deposited at Chair and Department of Pharmacognosy with Medicinal Plant Unit of the Medical University of Lublin in Poland.

### 3.2. Sample Preparation and Alkaloid Extraction

For each experiment a ground powder of 7.0 g of the dried plant material was placed in a special preparative 34 mL extraction cell. Additionally, 2 g of Celite (deactivate adsorbent) were added to maintain a constant temperature and seal the cell. Extraction of alkaloids was performed by PLE at elevated temperature of 140 °C and 100 bars pressure using MeOH as polar solvent using Dionex ASE 100 extractor in three cycles, 10 min each.

### 3.3. Method Optimization of VLC

Each experiment began with the incorporation of the sorbents onto glass column or polypropylene cartridge under vacuum followed by conditioning of filled column to activate it before applying the analyzed sample. For this purpose, a properly optimized solvent system was passed through the column. It is very important not to allow the column to dry between conditioning and applying the analyte. After that, concentrated methanolic extracts after removal of lectins by filtration have been transferred into glass column or polypropylene cartridge filled with different combinations of two polar sorbents: silica gel 60 F_254_ and basic Al_2_O_3_ (150 MeSh) as two-stationary phase gradient systems in various proportions and different order aiming to isolate alkaloidal fractions. VLC was carried out with different solvent gradient systems using: CHCl_3_, MeOH, Me_2_CO, 25% aqueous ammonia solution in various proportions ending to 90:5:5:0.1 (*v/v/v/v*). The first experiment was performed using a glass column (height 70 cm, diameter 2.5 cm) half-filled (about 20 cm of silica gel and 20 cm Al_2_O_3_) in equal parts of silica gel (25 g) and Al_2_O_3_ (57 g). In two subsequent further tests, one polypropylene cartridge (height 17.5 cm and diameter 3 cm) was used, alternately filled with Al_2_O_3_ and silica gel in a volume ratio of 1:3, whereas the last one in 3:1 volume ratio. Fractions of 20 mL were collected by rinsing the analytes with two 300 mL portions of the mobile phase consisting of CHCl_3_, MeOH, Me_2_CO and 25% aqueous ammonia solution (90:5:5:0.1, *v/v/v/v*). Then, elutions were carried out using 100 mL of MeOH:CHCl_3_ (1:1, *v/v*), and finally the columns were rinsed with 50 mL of MeOH solution. In total, 82 fractions were obtained: 34 from the first experiment, 31 from the second one and 17 from the third one. The obtained fractions were analyzed by TLC method on silica gel plates with the following standards used for comparison and confirmation of the presence of the Amaryllidaceae alkaloids (AAs): sanguinine, lycoramine, *nor*-galanthamine and galanthamine. Developed in the same VLC system and then identical fractions were collected together on the basis of their TLC profile. The separated alkaloids were visualized under UV light.

### 3.4. LC-MS Identification of the Isolated Compounds

The separation samples were analyzed by an HPLC/ESI-QTOF-MS system in positive ion mode using a 6530B Accurate-Mass-QTOF-MS mass spectrometer with an ESI-Jet Stream^®^ ion source and Atlantis HILIC silica column (150 × 2.1mm, *d*p = 3μm) (Waters Milford, MA, USA). The liquid chromatography system was equipped with DAD (diode array detector), autosampler, binary gradient pump, and column oven. Gradient of solvents: acetonitrile (95%) with 10 mM ammonium formate (0.2%) (solvent A) and acetonitrile (50%) with 10 mM ammonium formate (0.2%) (solvent B) was used as the mobile phase. The following gradient procedure was adopted: 0–10 min, 100% using solvent A; 10–40 min, 92% A and 8% B; 40–45 min, 64% A and 36% of solvent B. ESI-QTOF-MS analysis was performed according to the following parameters: total time of analysis was 45 min, with a stable flow rate at 0.25 mL/min. Injection volume for analyzed extracts was 10 μL. Dual spray jet stream ESI, (+)-positive ion mode, gas (N_2_) flow rate: 12 L/min., nebulizer pressure: 35 psig, vaporizer temp.: 300 °C; *m/z* range 100–1000 mass units, with acquisition Mode AutoMS^2^, collision induced dissociation (CID): 0, 10, 40 and 60 eV with MS scan rate 1 spectrum per s, 2 spectra per cycle, skimmer: 65 V, fragmentor: 150 V and octopole RF Peak: 750 V.

### 3.5. TLC with Bioautography of Anticholinesterase Activity

Detection of AChE inhibitory activity was carried out using the modified Fast Blue B Salt method. Bioautography was performed on TLC plates (0.2 thickness) covered with silica gel developed in an optimized system as a mobile phase (CHCl_3_, MeOH, Me_2_CO, 25% aqueous ammonia solution 90:5:5:0.1, *v/v/v/v*), containing an additional optimal concentration of 2-naphthylacetate (30 mg/20 mL) in the mobile phase. It was prepared in a separating funnel, mixed all together and taking lower organic phase for the plate development. The subsequent analysis took place in the Camag Derivatizer, which is an automated sprayer for derivatization of TLC plates. The developed and dried plates were transferred into the Derivatizer, where they were sprayed with acetylcholinesterase enzyme (3 U/mL) in TRIS buffer (50 mM, pH 7.8) stabilized with bovine serum and next incubated for 15 min. at 37 °C in the chamber. Then, after the incubation of the enzyme, Fast Blue B Salt water solution (1.25 mg/mL) was sprayed into the plates. After about few minutes white inhibition zones could be easily detected on a deep violet background.

## 4. Conclusions

The results obtained in research mentioned above clearly shows, that *Narcissus ‘Hawera’* among the others plants from genus *Narcissus* and, generally, Amaryllidaceae family is very rich source of bioactive alkaloids. From bulbs of *Narcissus triandrus L. c.v. ‘Hawera’*, several alkaloids with strong bioactivity were isolated using the innovative method. The gVLC method proved to be an efficient technique for purifying sample, concentration, fractionation and preparing for subsequent analysis. Preparation of the column did not require too much work and time, and the sorbents used proved to be suitable for the initial purification of plant fractions. This allowed us to shorten the duration of the analysis, by omitting some of processes like centrifugation and distillation. Only few published papers dealt with the application of VLC in separation of the Amaryllidaceae alkaloids [20,21,30], and only silica as the stationary phase was used. VLC was also a part of combined chromatographic methods, including also PLC and GC/MS. They were used, rather than for isolation of alkaloids, not for prefractionation and bioassays involving TLC-bioautographic or HPLC/MS analysis. No efficiency related to isolation of the bioactive alkaloids has been described there. Our three above-mentioned experiments allowed to obtain relatively pure fractions. The first experiment carried out on a glass column required large amounts of sorbent and it was time and solvent consuming. Moreover, preliminary evaluation of the fractions on the TLC plates showed that the initial fractions A1-A18 from the first experiment contained huge amounts of chlorophyll. Therefore, it was necessary to discard these factions. The remaining two experiments turned out to be much more efficient, less solvent and time consuming and permitted obtaining more fractions with potent AChE inhibitors present in higher amounts in the fractions. This method can be regarded as environmentally friendly due to lower usage of solvents. It gives highly reproducible results, and it can be easily combined with other techniques such as HPLC/ESI-QTOF-MS and TLC-bioautography. Satisfactory results have become the basis for shortening the duration of isolation of pure bioactive alkaloids for biological in vivo tests, but on the other hand, the obtained bioactive fractions will be also studied in in vivo tests, in the future. More and more often, modern medicine, disappointed with synthetic medicine, reaches for nature and the richness of the world of plants to find an antidote for the treatment of neurodegenerative diseases. The combined preparative and analytical approach presented here—PLE-gVLC-bioTLC-HPLC-ESI-QTOF-MS—in searching for bioactive *Narcissus* alkaloids has been described for the first time. It can speed up the whole procedure of the isolation of bioactive alkaloids for biological activity. Such improvements are important steps in the constant attempts to find medicine that would be unrivaled in the treatment of AD [31].

## Figures and Tables

**Table 1:** Fractions (30 in total) obtained in experiments using a different combination of sorbents by the VLC method.

The Number of Experiments	1-A	2-B	3-C
Type of column	glass	polypropylene cartridge	polypropylene cartridge
Sorbent filling ratio (Al_2_O_3_(150 MeSh): silica gel (60 F_254_))	1:1 Al_2_O_3_(25 g): silica gel (57 g)	1:3 Al_2_O_3_(17 g): silica gel (25 g)	3:1 Al_2_O_3_(57 g): silica gel (8 g)
Fractions obtained	A-19 A-20-22 A-23-24 A-25 A-26 A-27-33 A-34	B-3 B-4-7 B-8-9 B-10 B-11 B-12 B-13-14 B-15-17 B-18-20 B-21 B-22-25 B-26 B-27-29 B-30 B-31	C-2 C-3-4 C-5-8 C-9 C-10-12 C-13-14 C-15-16 C-17

A19-A34,B3-B31, C2-C17 are individual 50 mL fractions obtained from three performed experiments, as well as after discarding the initial fraction containing ballast substances (chlorophyll) and combining similar fractions based on their TLC profiles.

**Table 2:** Results of the eight active AChE inhibitors identified in the first experiment in the fractions A-19-34 by HPLC/ESI-QTOF-MS.

% of Isolated Compounds to the Total Amount of All Alkaloid Compounds Obtained from Individual Fractions [%]	Fraction Number						
	A-19	A-20-22	A-23-24	A-25	A-26	A-27-33	A-34
Sanguinine	1.2						
Lycoramine	54.3	9.9			27.5	16.4	5.6
Lycorine	7.2	52.9	41.2	23.6	17.8	10.0	10.0
Ungeremine		9.9	16.0	16.8	18.6	22.6	14.6
4,*N*-didehydro-*nor*-augustamine		17.7	15.5	7.2	15.9	18.1	11.9
Tetrahydro-*nor*-augustamine		4.7	10.7	8.8	12.5	21.4	16.6
Mesembrinole			6.2	7.1			24.6
Galanthamine					1.4	3.3	

**Table 3:** Results of twelve active AChE inhibitors identified in the second experiment in the fractions B10-31 by HPLC/ESI-QTOF-MS.

% of Isolated Compounds to the Total Amount of All Alkaloid Compounds Obtained from Individual Fractions [%]	Fraction Number											
	B-10	B-11	B-12	B-13-14	B-15-17	B-18-20	B-21	B-22-25	B-26	B-27-29	B-30	B-31
Sanguinine					3.4	9.2	9.1	5.8	7.5		1.4	2.9
Lycoramine	39.0	38.1	36.9									
Lycorine		11.6	26.6	29.2	22.6	17.1	21.2	22.2	22.8	26.1	9.1	
Ungeremine		7.7	4.5	5.8	9.2	7.6	7.2	6.3	11.5	9.2	24.9	26.1
4,*N*-didehydro-*nor*-augustamine		10.8	4.7	6.6	6.0	3.6	5.9	4.0	8.1			
Tetrahydro-*nor*-augustamine		5.8	3.4	4.3	4.7		0.8	0.8				
Mesembrinole					27.4	15.6	12.1	9.0	4.6			9.7
Haemanthamine				3.5								
Lycorine-*N*-oxide			0.7									
Galanthamine-*N*-oxide			2.1	1.6								
Galanthamine							1.9		1.7			
Tazettine							3.4	8.3	9.9	5.7		

**Table 4:** Results of eleven active AChE inhibitors identified in the third experiment of fraction by HPLC/ESI-QTOF-MS.

% of Isolated Compounds to the Total Amount of All Alkaloid Compounds Obtained from Individual Fractions [%]	Fraction Number									
	C-2	C-3-4	C-5-8	C-9	C-10-12	C-13-14	C-15-16	C-17	C-18-24	C-25
Sanguinine	1.7							8.7		
Lycoramine		7.5	26.5	53.5						
Lycorine	23.8	23.3	2.4		20.2	25.4	22.1	15.6	30.1	7.8
Ungeremine	4.2	8.2	6.7	4.1	4.1	10.3	11.2	7.8	2.0	10.0
4,*N*-didehydro-*nor*-augustamine					4.2	9.7	12.9	4.3		
Tetrahydro-*nor*-augustamine		5.5			2.2	3.3	2.8	1.3	9.3	3.7
Mesembrinole		6.7			13.4	36.2	10.3	4.3		
Galanthamine				5.9						
Lycoramine-*N*-oxide					2.1	2.4				
Haemanthamine					12.2	6.9	3.4	2.6		
Lycorine-*N*-oxide							2.8			
